# Taxonomic and functional diversity of cultured seed associated microbes of the cucurbit family

**DOI:** 10.1186/s12866-016-0743-2

**Published:** 2016-06-27

**Authors:** Eman M Khalaf, Manish N Raizada

**Affiliations:** Department of Plant Agriculture, University of Guelph, Guelph, N1G 2W1 ON Canada; Department of Microbiology and Immunology, Faculty of Pharmacy, Damanhour University, Damanhour, Egypt

**Keywords:** Evolution, Cucurbit, Endophyte, Seed, Microbiota, *Cucumis*, *Citrullus*, *Cucurbita*, *Lagenaria*, *Luffa*, *Human gut microbiome*

## Abstract

**Background:**

Endophytes are microbes that colonize plant internal tissues without causing disease. In particular, seed-associated endophytes may be vectors for founder microbes that establish the plant microbiome, which may subsequently contribute beneficial functions to their host plants including nutrient acquisition and promotion of plant growth. The *Cucurbitaceae* family of gourds (e.g., cucumbers, melons, pumpkin, squash), including its fruits and seeds, is widely consumed by humans. However, there is limited data concerning the taxonomy and functions of seed-associated endophytes across the *Cucurbitaceae* family. Here, bacteria from surface-sterilized seeds of 21 curcurbit varieties belonging to seven economically important species were cultured, classified using 16S rRNA gene sequencing, and subjected to eight in vitro functional tests.

**Results:**

In total, 169 unique seed-associated bacterial strains were cultured from selected cucurbit seeds. Interestingly, nearly all strains belonged to only two phyla (*Firmicutes*, *Proteobacteria*) and only one class within each phyla (Bacilli, γ-proteobacteria, respectively). *Bacillus* constituted 50 % of all strains and spanned all tested cucurbit species. *Paenibacillus* was the next most common genus, while strains of *Enterobacteriaceae* and lactic acid bacteria were also cultured. Phylogenetic trees showed limited taxonomic clustering of strains by host species. Surprisingly, 33 % of strains produced the plant hormone, indole-3-acetic acid (auxin), known to stimulate the growth of fruits/gourds and nutrient-acquiring roots. The next most common nutrient acquisition traits in vitro were (in rank order): nitrogen fixation/N-scavenging, phosphate solubilisation, siderophore secretion, and production of ACC deaminase. Secretion of extracellular enzymes required for nutrient acquisition, endophyte colonization and/or community establishment were observed. *Bacillus* strains had the potential to contribute all tested functional traits to their hosts.

**Conclusion:**

The seeds of economically important cucurbits tested in this study have a culturable core microbiota consisting of *Bacillus* species with potential to contribute diverse nutrient acquisition and growth promotion activities to their hosts. These microbes may lead to novel seed inoculants to assist sustainable food production. Given that cucurbit seeds are consumed by traditional societies as a source of tryptophan, the precursor for auxin, we discuss the possibility that human selection inadvertently facilitated auxin-mediated increases in gourd size.

**Electronic supplementary material:**

The online version of this article (doi:10.1186/s12866-016-0743-2) contains supplementary material, which is available to authorized users.

## Background

Plants as metaorganisms are associated with diverse microbes spanning different niches (such as rhizosphere, phyllosphere and endosphere), located within or on vegetative organs (such as roots, stems and leaves) and reproductive organs (such as flowers, fruits and seeds) of the host plant [1–4]. With respect to the endosophere, the term endophyte refers to microbes that reside in internal tissues of plants without showing any visible adverse effects on their host [5–7]. Either culture-dependent and/or -independent techniques have revealed the diversity of bacterial endophytes that encompass various bacterial taxa across a wide-range of different plant species [4]. Evidence suggests that endophytes originate from the rhizosphere (soil) and/or are maternally transmitted to future generations (e.g., vertical transmission through seeds) [8, 9].

The hologenome theory of evolution postulates that the host and its associated beneficial microbiota co-evolve as one unit to provide benefits to one another including nutrient acquisition, growth promotion and defense [10]. In particular, bacteria, including endophytes, can assist with plant nutrient acquisition through biological nitrogen fixation, mineral phosphate solubilisation and production of siderophores [7, 11, 12]. Several endophytes have the capacity to stimulate plant growth either directly or indirectly. The direct route involves microbial production of phytohormones such as auxin (indole-3-acetic acid, IAA) which is a major plant growth stimulator, especially for roots [12]. The indirect route includes the reduction of levels of the plant stress hormone, ethylene, which signals suppression of plant growth to conserve nutrients. Microbes can degrade the precursor of ethylene (ACC) via the enzyme, 1-aminocyclopropane-1-carboxylic acid (ACC) deaminase, resulting in stimulation of plant growth, especially roots [13].

The above nutrient and plant growth activities require colonization and community formation on plant surfaces [14]. For bacterial penetration into plant tissues, microbes secrete plant cell wall hydrolyzing enzymes such as pectinases and cellulases [7]. Microbial community formation can be facilitated by biofilms [14]; the biofilm matrix is composed of extracellular polymeric substances (EPS) comprising polysaccharides, proteins, nucleic acids and lipids. Biofilms confine microbially-secreted extracellular enzymes such as proteases (protein degrading enzymes), endocellulases and chitinases (polysaccharide degrading enzymes). These enzymes are responsible for breaking down their biopolymer substrates as energy sources [15].

Aside from potential impacts on their host plant, seed associated microbes play a substantial role in the plant life cycle. First, they can assist with seed health and make them ready for germination [16]. Second, these microbes can act as founders of the bacterial communities of the newly developed plant where they can provide benefits to their hosts. Most important, for multi-generational maintenance of these beneficial associations, plants can also use seeds as microbial vectors [3].

**Fig. 1:**
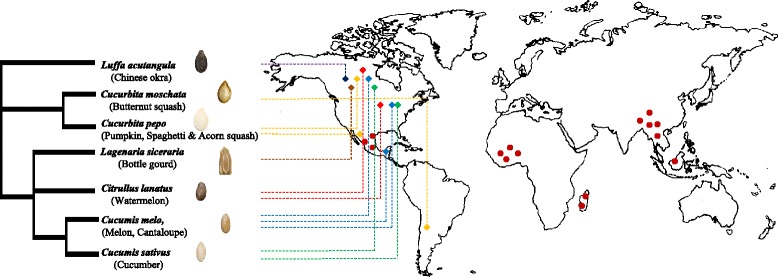
Geographical origins of the economically important cucurbits used in this study as sources of endophytes. Triangles indicate the immediate seed sources. Dark red circles indicate the hotspots of diversity of the *Cucurbitaceae*. The phylogenetic tree was constructed using PhyloT phylogenetic tree generator, based on NCBI taxonomy [29, 30]

The gourd family *Cucurbitaceae* is classified into 15 tribes encompassing 97 genera and 940–980 species [17]. Their highest diversity is observed in tropical and subtropical climates, with hotspots in Southeast Asia, West Africa, Madagascar, and Mexico (Fig. 1). However, some species can grow in temperate regions [17–19]. The family became nutritionally and culturally important to humans more than 12,000 years ago [20]. Today, based on their economic importance, three genera are of significant value within this family: *Cucumis*, *Citrullus*, and *Cucurbita,* representing commonly consumed species such as *Cucumis sativus*.L (cucumber), *Cucumis melo*. L (melon), *Citrullus lanatus* (Thunb.) Matsumura & Nakai (watermelon), and *Cucurbita* L. (squash and pumpkin), in addition to regionally important genera such as *Luffa* and *Lagenaria* [19, 21, 22]. According to recent taxonomic studies of the gourd family conducted by Schaefer and Renner [19], these five genera belong to three tribes (Table 1): Benincaseae [includes three genera; *Citrullus*, *Lagenaria* (*n* = 11) and *Cucumis* (*n* = 7 or 12)], Cucurbiteae [includes the genus *Cucurbita* (*n* = 20)] and Sicyoeae [including the genus *Luffa* (*n* = 13)]. Molecular studies based on chloroplast gene, spacer and intron sequences, suggest an Asian origin of the *Cucurbitaceae* family, followed by 43 long distance dispersal events over a 60 million year period to different continents [23].

**Table 1:** Summary of cucurbit seeds used as sources of endophytes in this study

Taxonomy^19^	No. of diploid chromosomes	Common name	Commercial variety	Source	Common use
Tribe Benincaseae					
Genus *Cucumis*					
* Cucumis melo* L.	24	Melon	Cantaloupe Delicious	Mckenzie, MB, Canada, #101114	Food
			Cantaloupe^a^	Honduras	
			Santa Claus cantaloupe^a^	USA	
			Canary cantaloupe^a^	USA	
			Honeydew^a^	USA	
* Cucumis sativus* L.	14	Cucumber	Field cucumber^a^	Canada	Food
			Straight eight	Mckenzie, MB, Canada, #101250	
			Marketmore (Certified organic seeds)	Mckenzie, MB, Canada, #134129	
			Spacemaster	Mckenzie, MB, Canada, #132403	
			Burpless beauty	Burpee, PA, USA #52175A	
			Burpless F1	Mr. Fothergill’s, AB, Canada, #13194	
Genus *Citrullus*					
* Citrullus lanatus (Thunb). Matsum.&Nakai*	22	Watermelon	Early Canada improved	Mckenzie, MB, Canada, #101734	Food
			Crimson sweet	Burpee, PA, USA, #62679A	
			Watermelon^a^	Canada	
Genus *Lagenaria*					
* Lagenaria siceraria (Molina) Stand.*	22	Bottle gourd	Bottle gourd	Ontario Seed Co., ON, Canada	Ornamental
Tribe Sicyoeae					
Genus *Luffa*					
* Luffa acutangula* (L.) *Roxb.*	26	Angled Loofah	Chinese okra	Stokes Seeds Ltd, ON, Canada Stokes Seeds INC., NY, USA, #422Y	Food
Tribe Cucurbiteae					
Genus *Cucurbita*					
* Cucurbita moschata Duch.ex Poir*	40	Squash & pumpkin	Butternut squash^a^	Argentina	Food
* Cucurbita pepo* L.	40	Squash & pumpkin			Food & ornamental
* C.pepo* L. *var. pepo* L.			Jack O’Lantern	Mckenzie, MB, Canada, #101556	
			Pumpkin^a^	Canada	
* C.pepo* L. *var. turbinate*			Acorn squash^a^	Mexico	
* C.pepo* L. *var. fastigata*			Spaghetti squash^a^	Mexico	

^a^Fresh product

^19^Taxonomic classification is derived from Schaefer and Renner, 2011

Despite the enormous diversity and economic importance of the *Cucurbitaceae* family to humans and the importance of seeds as vectors for endophytes, we could find few reports that focused on the discovery of seed bacterial endophytes across this nutritionally important plant family. Recently, the diversity of bacterial endophytes colonizing seeds (spermosphere), roots (endorhiza), flowers (anthosphere) and fruits (carposphere) of three different pumpkin cultivars was reported, along with potential applications to combating pumpkin diseases [24, 25]. A related publication by Glassner et al. [4] did not focus on seeds, but rather examined the culture-independent microbial communities (via *in-situ* visualization) and culturable microbial communities associated with fruit flesh tissues and placenta (seed cavity without seeds) of selected species and/or varieties of melon, wild melon, watermelon, colocynth and bryony [4].

In contrast to the *Cucurbitaceae*, there have been many reports of the seed associated microbiome across a diversity of plants including gymnosperm trees, legumes, cereals, fruits and vegetables [3]. For example, the diversity of seed associated bacterial endophytes in rice has been reported across two consecutive generations [26], This research also demonstrated that changes in host physiology can cause coincident changes in the composition and diversity of the seed endophytic bacterial communities. In the genus *Zea*, which includes corn (maize), it was demonstrated that seed endophytic communities co-evolved with their hosts, with a core microbiome that was conserved across domestication, migration and breeding events [27]. Similarly, by comparing the seed surface microbiomes of different geographic samples of wheat (*Triticum* spp.) and canola (*Brassica* spp.), a conserved epiphytic microbiome was identified [28].

The objectives of this study were to investigate the conservation and diversity of the culturable microbiome of seeds of seven economically important *Cucurbitaceae* species, including various varieties (21 in total), with respect to endophytic taxonomy and activities related to plant growth promotion and nutrient acquisition.

## Methods

### Sources of cucurbit seeds

The seeds of 21 cucurbit varieties, representing seven species and five genera [29, 30] were used in this study as sources of endophytes (Table 1, Fig. 1). Commercial dry seeds and fresh fruits were purchased from different seed companies and local grocery stores in Ontario, Canada, respectively (Table 1). Fresh melons and squash were imported from different countries in North, Central and South America.

### Surface sterilization of dried commercial seeds

Seeds were surface sterilized using modifications of a frequently used protocol, adjusted for the varying texture of cucurbit seeds [7]. Per variety, three replicate pools of seeds were surface sterilized. For each replicate, 15 seeds per variety were surface sterilized using sodium hypochlorite (2.5–3.5 %) for 5 min (8 min for Jack O’Lantern pumpkin seeds) based on differences in the texture of the seed coat, then the bleach was drained. This step was performed twice, and then the seeds were rinsed with autoclaved distilled water, before being washed with 95 % ethanol for 5 min. After draining the ethanol, the seeds were rinsed three times for 5 min with autoclaved distilled water. To verify that the surface sterilization was adequate, 200 μl of the last wash were plated and cultured on three different types of agar media: LGI agar to capture contaminating diazotrophic bacteria [25 g sucrose, 0.01 g FeCl_3_•6H_2_O, 0.8 g K_3_PO_4_, 0.2 g MgSO_4_•7H_2_O, 0.2 g CaCl_2,_ 0.002 g Na_2_MoO_4_•2H_2_O, agar 15 g/l, pH 7.5], potato dextrose agar (PDA) (#70139, Sigma) to culture copiotrophic bacteria and fungi, and Reasoner´s 2A agar (R2A) (#17209, Sigma) to capture oligotrophic bacteria. Plates were incubated at room temperature for 3 days.

### Surface sterilization of fresh fruits for extraction of seeds

Three fruits were used for each variety representing three replicate pools of seeds. The fruits were first washed with soap and water, then heavily sprayed with 70 % ethanol under sterile conditions and left to dry. Fifteen fresh seeds were extracted from each fruit under aseptic conditions using sterile and/or autoclaved tools. The seeds were rinsed with autoclaved distilled water for seven to ten times (based on differences in the fibres and flesh coating of the seeds) until the surrounding fibers and mucilage were removed. Finally, 200 μl of the last washes were cultured on the same agar media used for verification of surface sterilization of commercial seeds to ensure null microbial growth.

### Isolation of bacterial endophytes

Once seed surface sterility was confirmed, 15 seeds/replicate were ground gently in an autoclaved mortar using 0.5 ml of 50 mM Na_2_HPO_4_ buffer per gram of seed dry weight [27]. The ground seed suspension (100 μl) was used for microbial culturing: 10-fold serial dilutions in 50 mM Na_2_HPO_4_ (10X, 100X and 1000X) were streaked on R2A, PDA and LGI agar plates (described above) followed by incubation for 1–7 days at 28 °C. Morphologically unique bacterial colonies from each plate were selected, streaked on fresh plates to purify and finally cultured in LB broth (10 g/L tryptone, 5 g/L yeast extract and 5 g/L NaCl, pH 7.2) for glycerol stocks and DNA isolation.

### Bacterial endophyte 16S rRNA gene fingerprinting

For identifying bacterial endophytes, DNA was isolated using Qiagen kits (#51306, QIAamp® DNA Mini kit) and then quantified (Nanodrop, Thermo Scientific, USA). PCR was performed using 16S rRNA gene universal primer pair [31], 799 F (5′-AACMGGATTAGATACCCKG-3′) and 1492r (5′-GGTTACCTTGTTACGACTT-3′). Approximately 50 ng of total DNA was added to a PCR mixture containing 4 μl Standard Taq Buffer (M791B, Promega), 0.4 μl of 25 mM dNTP mix, 0.5 μl of each primer (10 mM working stock), 0.6 μl of 50 mM MgCl_2_, 0.2 μl of Standard Taq (New England Biolabs) and H_2_O to a final volume of 20 μl. Following an initial denaturation at 94 °C for 7 min, DNA was amplified using 34 PCR cycles in a PTC200 DNA Thermal Cycler (MJ Scientific, USA) at 94 °C for 45 s, 48 °C for 1 min, 72 °C for 2 min, and a final extension at 72 °C for 7 min. Amplicons of 693 bp size were gel purified using the GFX™ PCR DNA and Gel Band purification kit (#45001489, GE Healthcare). Purified amplicons were sequenced using standard BigDye reaction conditions with an annealing temperature of 50 °C (3730 DNA analyzer, Applied Biosystems, USA). Reads were searched against bacterial 16S entries in RDP (Ribosomal Database Project) [32] using a 95 % confidence level, and further searched using BLASTN using default parameters. To generate maximum likelihood (ML) phylogenetic trees with bootstrapping of 500 replicates, all 16S sequences were trimmed and edited using Bioedit software [33], then aligned and used to generate the trees using default settings of MEGA6 software [34].

### Characterization of bacterial endophytes

The endophytes were subjected to eight in vitro tests. All experiments were performed in triplicate. Bacterial endophytes were initially preserved as glycerol stocks in 96-well plates with adjusted OD_600_ ≈ 0.4-0.6. Prior to each in vitro test, 96-well LB broth plates (1 ml/well) were inoculated from glycerol stock plates and incubated overnight at 37 °C with shaking. These cultures were then used to inoculate the media described below using a flame-sterilized 96 pin replicator:

### Growth on nitrogen free LGI media

All bacterial isolates were tested for their ability to fix and/or scavenge nitrogen [27, 35]. Prior to media preparation, all glassware was cleaned with 6 M HCl. An autoclaved 96 deep-well plate (2 ml well volume) was pipetted with 1 ml/well of sterile LGI broth [25 g sucrose, 0.01 g FeCl_3_•6H_2_O, 0.8 g K_3_PO_4_, 0.2 g MgSO_4_•7H_2_O, 0.2 g CaCl_2_ and 0.002 g Na_2_MoO_4_•2H_2_O, pH 7.5]. Following bacterial inoculation with the pin replicator, the plate was sealed with a sterile breathable membrane, incubated at 28 °C with gentle shaking, and OD_600_ readings were taken after 5 days.

### 1-aminocyclopropane-1-carboxylate (ACC) deaminase activity

Bacterial endophytes were screened for growth in the presence of ACC as their only source of nitrogen [27]. All glassware was cleaned with 6 M HCl before media preparation. 2 M ACC (#A3903,Sigma) was prepared in water and filter sterilized. Autoclaved LGI broth was amended with ACC solution to a final concentration of 1 μl/ml, and distributed in a new 96 deep-well plate (as above). Following bacterial inoculation with the pin replicator, breathable membranes were used to seal plates, and then the plates were incubated at 28 °C with gentle shaking. After 5 days, OD_600_ readings were taken, and only wells with a significant increase in OD than their corresponding nitrogen-free LGI wells were scored as ACC deaminase producers.

### Mineral phosphate solubilization

For detecting phosphate solubilizing microbes, Pikovskaya’s agar [36] was inoculated using the pin replicator. The media was prepared as follows: 0.5 g/L yeast extract, 10 g/L glucose, 5 g/l Ca_3_PO_4_, 0.5 g/l (NH4)_2_SO_4_, 0.2 g/l, KCl, 0.1 g/l MgSO_4_, 0.0001 g/l MnSO_4_, 0.0001 g/l FeSO4•7H_2_O, and 15 g/l agar, pH 7.2, then autoclaved (glucose was first filter sterilized and added after autoclaving). After incubation for 3 days at 28 °C in darkness, clear halos around colonies indicated the ability of microbes to solubilize tricalcium phosphate.

### Production of auxin (indole-3-acetic acid, IAA)

Using the 96 pin replicator, bacterial endophytes were plated on autoclaved LB agar (10 g/l tryptone, yeast extract 5 g/l, 5 g/l NaCl and 12 g/l agar, pH 7.2) supplemented with L-tryptophan to a final concentration of 5 mM [37]. The plates were incubated at 28 °C for 3 days, then overlaid with a pure nitrocellullose membrane, and kept overnight in the fridge at 4 °C, to allow the permeation of bacteria and their metabolites into the membrane. The next day, the nitrocellulose membrane was transferred to Whatman #2 filter papers impregnated in Salkowski reagent [2 % 0.5 M FeCl_3_ in 35 % perchloric acid (#311421, Sigma)] and left for 30 min. Dark pink halos around colonies in the membrane were interpreted as evidence for auxin production.

### Siderophore production

All glassware was deferrated with 6 M HCl and distilled, deionized water prior to media preparation [38]. In this deferrated glassware, LB agar media was prepared, autoclaved, poured into 150 mm Petri dishes and inoculated with bacteria using the 96 pin replicator. Plates were overlaid with O-CAS overlay after a 3 day incubation period at 28 °C [39]. For preparing 1 litre of O-CAS overlay, in deferrated glassware, a mixture consisting of 30.24 g of finely crushed piperazine-1,4-bis-2-ethanesulfonic acid (PIPES) with 10 ml of 1 mM FeCl_3_•6H_2_O in 10 mM HCl solvent, was added slowly with stirring to a mixture of 60.5 mg of chromeazurol S (CAS) and 72.9 mg of hexadecyltrimethyl ammonium bromide (HDTMA). Pre-warmed O-CAS was trapped in melted 1 % agarose in a proportion of 3:1 (v/v) just prior to pouring the overlay. The reaction was allowed to incubate for 15 min at room temperature, and then color changes were recorded by scoring purple halos as catechol-type siderophores and orange colonies as hydroxamate-type siderophores.

### Cellulase activity

To test the cellulase activity of bacterial isolates, R2A media was supplemented with 0.2 % (w/v) carboxymethylcellulose (CMC) sodium salt (C-8758, Sigma) and 0.1 % triton X-100 then autoclaved and poured into 150 mm plates [40]. Bacterial endophytes were inoculated using the 96 pin plate replicator, and then the plates were incubated in darkness at 28 °C for 3 days. Bacterial isolates displaying cellulase activity were visualized by flooding the plate with Gram’s iodine. Colonies surrounded by clear halos were interpreted as positive results.

### Pectinase activity

A previously published method was adapted [41] as described earlier [27]. R2A media amended with 0.2 % (w/v) of citrus pectin (P9135, Sigma) and 0.1 % triton X-100 was autoclaved and poured in 150 mm Petri plates. Following bacterial inoculation with the 96 well pin replicator, the plate was incubated at 28 °C for 3 days. The plate was then flooded with Gram’s iodine. Pectinase activity was scored as development of clear halos around colonies.

### Protease activity

Plates of tryptic soy agar/20 (twenty-fold diluted tryptic soy broth and 15 g/l agar) amended with 5 % (v/v) skimmed milk were inoculated using the 96 pin plate replicator into 150 mm Petri plates [42, 43]. The plates were incubated at 28 °C for 1–2 days, and positive colonies were scored as surrounded by clear halos.

## Results

### Taxonomic diversity of cultivated cucurbit seed-associated microbiota

**Fig. 2:**
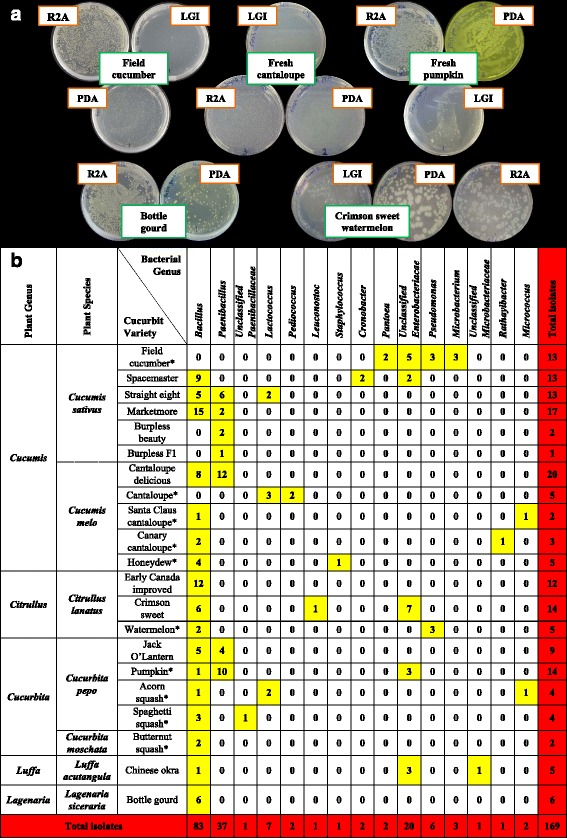
The collection of putative seed-associated endophytes cultured from cucurbits in this study. **a** Selected photos of cucurbit seed associated bacterial endophytes cultured on different agar media (R2A, LGI, PDA). **b** Distribution of isolated bacterial genera across tested cucurbit varieties. The yellow highlight refers to the number of isolates in each bacterial genus from a corresponding cucurbit variety. The asterisk denotes that the seed source is from fresh fruit

**Fig. 3:**
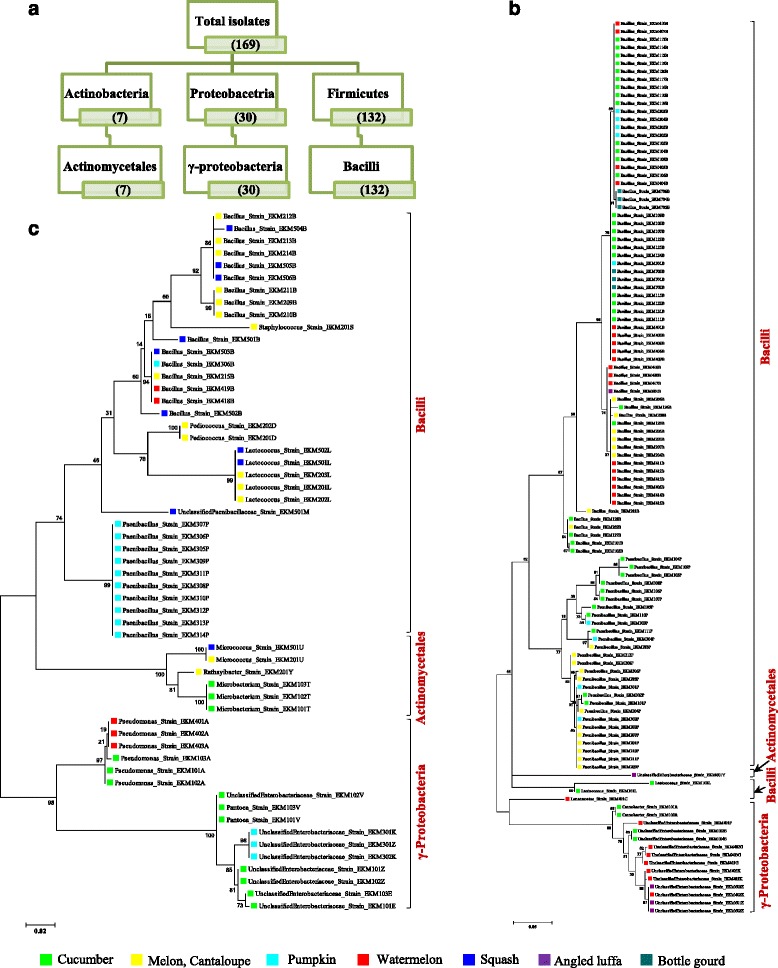
Maximum likelihood (ML) phylogenetic trees of putative seed-associated endophytes cultured from cucurbits based on bacterial 16S rRNA gene sequences. Bootstrap values are indicated above the branches. **a** Diagrammatic sketch of the taxonomy of the endophytes based on phylum and class/order. **b** ML tree of the endophytes from commercial cucurbit seeds (dry). **c** ML tree of the endophytes isolated from fresh fruit seeds

**Fig. 4:**
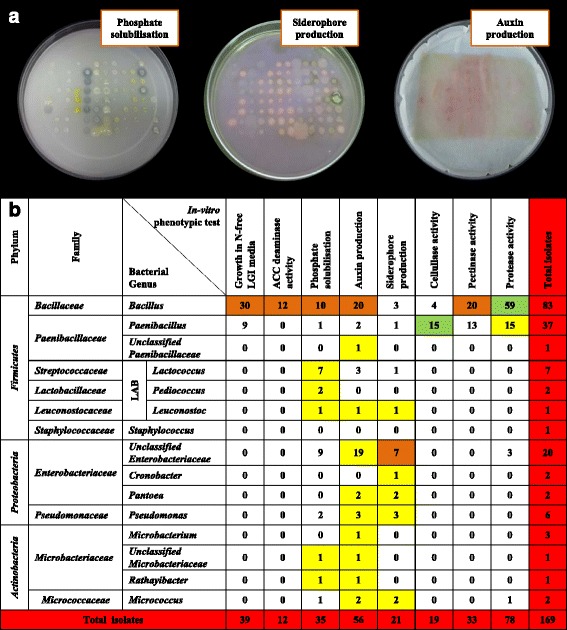
Summary of in vitro functional traits of putative seed-associated endophytes cultured from cucurbits, grouped by bacterial genus. **a** Selected photos for the in vitro phenotype screens used. **b** Distribution of in vitro functional traits of the endophytes grouped by bacterial genus. The yellow highlight refers to the most prevalent functional trait exhibited by the endophytes within a bacterial genus (row). The dark orange highlight refers to the most prevalent bacterial genus that displays a particular functional trait (column). The green highlight refers to the intersection of the two categories. LAB: lactic acid bacteria

From the tested curcurbit species and varieties (Table 1), a total of 169 unique seed-associated bacterial strains were cultured (Fig. 2). For taxonomic identification, V5-V9 regions of 16S rRNA genes were amplified (ranging in length from 334–682 bp) and searched using BLASTN and RDP databases. A total of 134 strains showed 100 % nucleotide sequence identity to the RDP database, 12 showed ≥ 95 % identity and 23 showed <95 % identity at a confidence level of 95 % (Additional file 1: Table S1). Best sequence matches from RDP classifier were used to create Maximum likelihood tree(s) (Fig. 3, Additional file 2: Figure S1). These sequences were deposited in Genbank and received the following accession numbers: [GenBank:KT220264, KT220265], [GenBank:KT222780-KT222785], [GenBank:KT281286-KT281446]. The phylogenetic composition of cultivated cucurbit seed-microbiota showed two significant findings: First, the cultured strains were predicted to belong to 15 bacterial genera within three phyla (*Firmicutes*, *Proteobacteria* and *Actinobacteria*) and within each phyla, all microbes belonged to one class/order only (class Bacilli, class γ-proteobacteria and order Actinomycetales, respectively) (Fig. 3a, Additional file 2: Figure S1). Second, the Bacilli class predominated across the five tested cucurbit genera, belonging to six different families (*Bacillaceae, Paenibacillaceae, Streptococcaceae, Lactobacillaceae, Leuconostocaceae* and *Staphylococcaceae*) (Figs. 3, 4b). Less common were two families of γ-proteobacteria (*Enterobacteriaceae, Pseudomonaceae)* and two families of *Actinobacteria* (*Microbacteriaceae and Micrococcoceae*).

### Conservation of cucurbit seed-associated microbiota

The genus *Bacillus* was the cultured core seed-associated microbiota, spanning the five tested cucurbit genera and accounting for ~50 % (83/169) of the total isolates (Fig. 2b), of which 80 strains showed 100 % sequence identity to *Bacillus* 16S rRNA genes in RDP, while the remaining 3 strains showed 99 % identity (Additional file 1: Table S1)*.* The strains were predicted to predominantly belong to diverse *Bacillus* species based on 16S rRNA gene BLASTN searches to Genbank such as *B. safensis*, *B. altitudinis*, *B. siamensis, B. pumilus* and *B. cereus* (Additional file 1: Table S1)*.* Strains of the genus *Paenibacillus* were the next most common (37/169), exclusively isolated from *Cucumis sativus* L*., Cucumis melo* L*.* and *Cucurbita pepo* L*. var pepo* L*.* Although members of *Enterobacteriaceae* family were less abundant (24/169), they showed higher taxonomic diversity on BLASTN searches (Fig. 2b), though the 16S rRNA gene sequence identities were often low (Additional file 1: Table S1)*.* Interestingly, three different genera of lactic acid bacteria (LAB) were recovered (10/169) from *C. sativus* L*., C. melo* L*.*, *Citrullus lanatus var. lanatus*. and *Cucurbita pepo* L*. var. turbinata* (Fig. 2b, Additional file 1: Table S1).

### Clustering of *Bacillus* and *Paenabacillus* communities by host

Using 16S rRNA gene sequences, some clustering of microbes by host were observed for both commercial (Fig. 3b) and fresh seeds (Fig. 3c). The clustering pattern of strains belonging to the two most abundant genera, *Bacillus* and *Paenabacillus*, separated for commercial and fresh seed sources was noticeably demonstrated (Fig. 3). With respect to commercial varieties (Fig. 3b), the *Bacillus* strains isolated from cucumber generally clustered separately from melon; pumpkin and bottle gourd *Bacillus* strains co-clustered with cucumber. *Bacillus* strains isolated from commercial watermelon showed three clusters, a large group (containing two subgroups) that clustered with melon, and two smaller groups that clustered with cucumber. With respect to *Paenabacillus*, strains from commercial cucumber generally separated from commercial melon, while pumpkin strains were interspersed (Fig. 3b). Most pumpkin *Paenabacillus* strains from this study were from fresh seeds (Fig. 3c). Some microbial clusters spanned different varieties within a host species, and hence were not variety-specific.

### Host specific microbiota

It was a striking feature that we were able to culture only a single bacterial genus from the seeds of some cucurbit varieties (Fig. 2b, Additional file 1: Table S1). For instance, from fresh field cucumber seeds, only *Microbacterium* strains could be isolated; however other bacterial isolates were cultured from an early surface sterilization rinse that included residual placenta and fruit pulp tissues. Similarly, 100 % of strains cultured from seeds of Early Canada improved watermelon, butternut squash and bottle gourd were predicted to be *Bacillus*. By contrast, all isolates cultured from fresh cantaloupe were predicted to be lactic acid bacteria comprising two bacterial genera, *Lactococcus* and *Pediococcus* (Fig. 2b). On the other hand, the isolated bacterial endophytes from cucumber (*Cucumis sativus* L.) and angled luffa (*Luffa acutangula*) spanned the three phyla (*Firmicutes*, *Proteobacteria* and *Actinobacteria*). However, the identified isolates from other tested cucurbit species were restricted to one or two phyla (Additional file 2: Figure S1). For example, endophytic bacteria from watermelon (*Citrullus lanatus*) and pumpkin (*C. pepo L. var. pepo* L.) belonged to the phyla *Firmicutes* and *Proteobacteria.* Nevertheless, all bacterial isolates from bottle gourd exclusively belonged to *Firmicutes.*

### Functional diversity of cultivated cucurbit seed-associated microbiota grouped by bacterial genus

Bacterial endophytes were phenotypically tested in vitro for traits associated with plant nutrient acquisition (nitrogen, phosphorus, iron, proteases to scavenge amino acids), and plant growth promotion (ACC deaminase activity, auxin production) along with traits associated with bacterial colonization and community establishment (cellulase, pectinase, proteases) (Fig. 4, Additional file 3: Table S2). The most prevalent phenotypic traits were protease activity, auxin production, nitrogen fixation/N-scavenging (growth on LGI media) and phosphate solubilization, representing ~46 %, ~33 %, ~23 % and ~21 % of all isolates, respectively. The rarest trait was ACC deaminase (7 % of strains). Of the strains that might contribute N-fixation/N-scavenging activity to their hosts, 30/39 were from *Bacillus*. With respect to auxin producing strains, 20/56 were *Bacillus*, while 21/56 were from *Enterobacteriaceae*. Of the strains that may contribute ACC deaminase activity, 12/12 were from *Bacillus*. For siderophore activity, 10/21 strains were from *Enterobacteriaceae*. Most or all of the strains that showed protease, pectinase or cellulase activities belonged to *Bacillus* and *Paenibacillus*. Phosphate solubilization was more randomly distributed, though 10/10 of the lactic acid producing strains showed this activity. It is noteworthy, that combined, the *Bacillus* strains could contribute all of the traits to their hosts, and furthermore that 21/24 *Enterobacteriaceae* strains were auxin producers (Fig. 4b, Additional file 3: Table S2).

### Functional diversity of cultivated cucurbit seed-associated microbiota grouped by their host plants

**Fig. 5:**
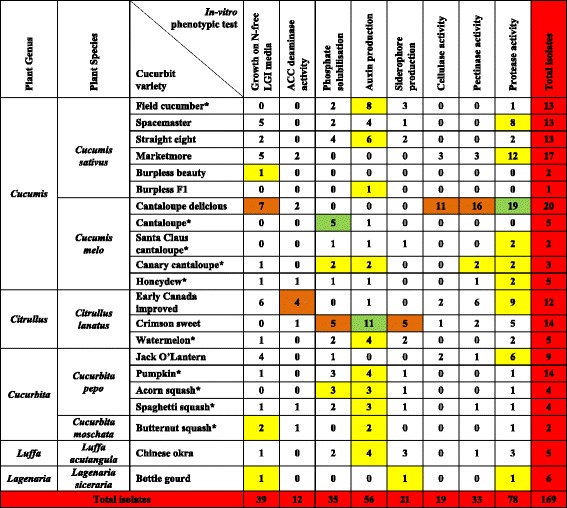
Summary of in vitro functional traits of putative seed-associated endophytes cultured from cucurbits, grouped by their cucurbit host plant. The yellow highlight refers to the most prevalent functional trait exhibited by the endophytes within a cucurbit variety (row). The dark orange highlight refers to the most prevalent cucurbit variety that displays a particular functional trait by their seed-associated endophytes (column). The green highlight refers to the intersection of the two categories. The asterisk denotes that the seed source is from fresh fruit

All endophytic phenotypic traits spanned all tested cucurbit species with a few exceptions for *Luffa acutangula* and *Lagenaria siceraria* but these were only represented by a single variety each (Fig. 5). For example, of the 56 strains that produced auxin, 19 were isolated from cucumber, 5 from melon, 16 from watermelon, 12 from pumpkin/squash, and 4 from luffa. A few varieties were inhabited by microbes that may have had biased phenotypic activities; for example, most of the seed-associated microbiota from Cantaloupe delicious produced extracellular enzymes. Of the 14 isolates from Crimson sweet watermelon, 11 were auxin producers. Of the 10 lactic acid bacteria (mineral phosphate solubilizers), 5 were isolated from fresh cantaloupe.

## Discussion

Seeds are critical vectors for diverse pathogenic and phyto-beneficial microbes including endophytes. There are three main routes of microbial transmission from mother to seeds: via the internal vascular system, foliar transmission through stigma, or direct contact with microbial communities on adjacent maternal organs such as fruits and flowers [44]. Physiological changes during seed maturation influence the functional properties of seed-colonizers and their taxonomic diversity. Following germination, the seed-associated endophytes have the potential to become the founders of the seedling microbiome, to provide beneficial functions to the host such as nutrient acquisition activities within the spermosphere and rhizosphere [3].

The *Cucurbitaceae* is one of the most economically important families of crops, used as food and for industrial and medical purposes [21, 45]. Up to 50 % of the weight of cucurbit seeds consists of oil, and another 35 % of protein, making them a richer source of nutrients than most cereals and legumes [46, 47] which has contributed to them being staple food sources in traditional societies [48–50]. The cucurbits were domesticated on diverse continents, and there is tremendous genetic variation within the family (Table 1) [22]. We hypothesized that the most critical cucurbit endophytes would be transmitted by seeds and would be conserved across the family, reflecting an ancient evolutionary origin, predating species diversification. From this study, a total of 169 seed-associated bacterial endophytes were cultured from 21 varieties representing seven different species of nutritious cucurbits. Despite this diversity, the results demonstrate that several economically important cucurbit species possess a core culturable, seed-associated microbiota, dominated by the genus *Bacillus* (Fig. 2). Based on in vitro testing, this study further demonstrates that the tested cucurbit seeds are potential vectors for microbes with diverse nutrient acquisition activities (Fig. 4, Fig. 5). These microbes were isolated primarily from internal seed tissues, and a limited number from the seed surface (fresh field cucumber).

### Phyla and class level diversity of seed associated endophytes across the tested cucurbits

This study represented seven species and 21 varieties of cucurbit seeds including dried seeds from diverse commercial sources, and fresh seeds from plants grown in diverse agro-ecological environments (Table 1). Despite this diversity, the putative endophytes belonged to only 15 bacterial genera within three phyla, with 7 genera from *Firmicutes*, ≥4 from *Proteobacteria*, and 4 from *Actinobacteria* on RDP searches (Fig. 2). These results are consistent with previous reports [3, 12], with a recent meta-analysis reporting that the majority of seed endophytes from diverse plant species belong to the phylum *Proteobacteria* (represented by 80 genera), especially γ*-proteobacteria* (41 genera), followed by *Actinobacteria* (25 genera) and *Firmicutes* (20 genera) [3]. Our study further shows that within each of the phyla (*Firmicutes*, *Proteobacteria*, *Actinobacteria*), the seed-associated endophytes of the cucurbits belong to one class/order only (class Bacilli, class γ-proteobacteria and order Actinomycetales, respectively) (Fig. 3a).

### Conservation and clustering of *Bacillus* and *Paenibacillus* across the tested cucurbits

As noted above, a striking feature of our study is that the genus *Bacillus* appears to comprise the culturable core seed microbiota across the five tested cucurbit genera. *Paenibacillus* was the next dominant genus, but culturable from only three of the cucurbit species (Fig. 2b)*.* Together, these two genera comprised 120/169 of the total isolated endophytes. We observed only limited clustering of *Bacillus* and *Paenibacillus* strains by host species (Fig. 3), which may suggest that the functional traits of the microbes may be as important as their taxonomy (see below). Both *Bacillus* and *Paenibacillus* are gram-positive, aerobic, endospore-forming bacteria, that inhabit different ecological niches such as plants, rhizosphere, soil and water [51]. Previous studies from other plants have shown that a characteristic feature of seed endophytes is that they are endospore forming; this property is thought to protect the seed inhabitants from changes within the seed (to tolerate storage, desiccation, seed maturation, germination) [3]. *Bacillus* strains, in particular, are important commercial biofertilizers and/or biopesticides used for crop production [52–54].

Both *Bacillus* and *Pseudomonas* have been shown to be the most dominant bacterial genera of the seeds of other plants, in addition to other genera such as *Paenibacillus*, *Micrococcus*, *Staphylococcus*, *Pantoea*, and *Actinobacter* [3]. Though *Bacillus and Paenibacillus* were common in the tested cucurbit seeds, these genera are rare in maize (*Zea*) seeds [27], suggesting that the cucurbits may have selected these genera for specific ecological purposes. We could find few relevant reports concerning cucurbit seed endophytes; however an early report from 1976 implied the isolation of *Bacillus* strains [55]. A more recent report aimed at developing a synergistic biocontrol strategy for pumpkin diseases, noted the isolation of anti-pathogenic strains of *Paenibacillus polymyxa* from pumpkin seeds, *Lysobacter gummosus* and *Pseudomonas chlororaphis* from roots and *Serratia plymuthica* from flowers [24], while a follow up study showed the potential for development of new inoculants [25]. In another study, fluorescence *in-situ* hybridization was shown to be an effective method to discover bacterial endophytes residing in the seed cavity of selected cucurbit species [4]. The study revealed the existence of α, β, γ- *proteobacteria*, *Firmicutes* and *Actinobacteria* inside the fruit, and the predominance of the genus *Bacillus* across the fruits that were tested.

In the present study, ~38 % (14/37) of *Paenibacillus* isolates from the tested cucurbits were associated with pumpkin seeds of which two strains were predicted to be *P. polymyxa*. The authors of the earlier pumpkin study also examined the microbiota of roots, flowers and fruits, and suggested the prevalence of two bacterial genera, *Pseudomonas* and *Bacillus,* that spanned all tested microenvironments, though the study focused on the anti-pathogenic properties of these strains not biofertilizer activity [24]. *Bacillus* strains have previously been isolated from different tissues of watermelon (*B. amyloliquefaciens, Bacillus subtilis, Bacillus* sp.) [56, 57] and Hami melon [58], from the rhizosphere and surface sterilized roots of cucumber [59, 60], and from the leaf surface and rhizosphere of different cucurbits [61]. In the future, it will be interesting to study whether these root, shoot, rhizosphere associated *Bacilli* originate from seeds after germination.

As noted above, *Bacillus* is a commonly used biopesticide [53, 54, 62] which might raise concerns that the strains isolated in this study are commercial inoculants; however, in our study design, we deliberately selected cucurbits that were not organic certified, with the exception of the cucumber variety Marketmore which was verified by the associated company as having not been treated with inoculants. Furthermore, we observed a large diversity of seed-associated *Bacillus* isolates (13 different *Bacillus* species based on BLASTN searches; Additional file 1: Table S1) from varieties grown in different geographic locations (Table 1), but we cannot rule out that some of these may have originated from spores deposited in the soil from commercial inoculants.

### Conservation and diversity of potential functional traits provided by cucurbit seed-associated microbiota

Several potential functional traits were conserved in the seed-associated microbiota across the tested cucurbits:

### Auxin production

In plants, the hormone auxin, specifically indole-3-acetic acid (IAA), is responsible for cell elongation, division and differentiation [63]. Auxin is also synthesized by bacteria, including *Pseudomonas*, *Enterobacter*, *Pantoea*, *Acinetobacter*, *Klebsiella*, *Bacillus*, *Agrobacterium*, *Azotobacter*, *Micrococcus* as well as *Paenibacillus polymyxa* [51, 63–65]. From this study, a striking feature was that 33 % of all seed-associated bacteria were auxin producers, including 21/24 strains of *Enterobacteriaceae,* 20/83 *Bacillus* strains, 3/6 *Pseudomonas* strains, and two *Paenibacillus* strains, including one putative *P. polymyxa* strain (Fig. 4). This result contrasts sharply with seeds from the *Zea* family, which includes corn, in which only 7 % of strains were auxin producers [27]. It is interesting to speculate whether humans inadvertently selected for auxin-producing microbes in cucurbit seeds, as they selected for larger fruits/gourds or more nutritional seeds. In tomato fruit, which is a model system for understanding the genetic regulation of fruit size, the locular cells that surround seeds undergo cell expansion which is associated with larger fruit size; examination of the tomato transcriptome suggested the role of auxin in this cell expansion [66]. Nutritionally, the amino acid tryptophan which is essential for humans, is the precursor for auxin (IAA) synthesis in plants [12]. In pre-Columbian North America, indigenous tribes grew the “three sister” crops, corn, beans and squash, in part as a source for tryptophan which is deficient in corn and low in beans [67, 68]. Squash was firstly domesticated in western Mexico around 10,000 years ago, followed by maize and then beans [69, 70]. Numerous studies note the value of cucurbit seeds, especially squash, pumpkin and watermelon, as sources of essential amino acids including tryptophan [68, 71–75]. In this study, ~70 % (39/56) of the auxin producers were indeed isolated from these three cucurbits (Fig. 5). It may be that as humans selected for more nutritional cucurbit seeds containing tryptophan, they facilitated microbial synthesis of auxin, which in turn contributed to increased gourd size - another favoured trait.

### ACC deaminase

Ethylene was originally isolated as the plant hormone that stimulates fruit-ripening. It is also an important stress hormone for plants to signal the onset of abiotic and biotic stress [76]. The enzyme ACC deaminase is secreted by diverse microbes and prevents synthesis of ethylene by converting ACC, the precursor of ethylene, into α-ketobutyrate and ammonia [12, 77]. In the present study, ACC deaminase activity was observed in only 7 % of strains, exclusively belonging to the genus *Bacillus* (Fig. 4), in contrast to previous studies [77] which showed that other genera (including *Burkholderia*, *Enterobacter*, and *Pseudomonas*) produce this enzyme, including 20 % of microbes associated with *Zea* seeds [27]. Plant growth promoting microbes with ACC deaminase activity are thought to assist plants by relieving the inhibitory effect of ethylene on root growth [13].

### Nitrogen fixation and/or scavenging

Plants form symbiotic relationships with microbes, including endophytes to facilitate biological nitrogen fixation (BNF), the conversion of atmospheric nitrogen gas into a usable form of nitrogen [12, 51]. In this study, 23 % of seed associated microbes could grow on N-free media (Fig. 4), suggestive of either BNF or N-scavenging; all of these strains were *Bacillus* or *Paenibacillus.* Diverse species of *Bacillus* and *Paenibacillus* have previously reported as nitrogen fixers [78–80]. In the future, we suggest testing these microbes *in planta* to verify whether they can contribute to improved nitrogen use efficiency in these crops.

### Phosphate solubilization

Phosphorous is the second most limiting nutrient after nitrogen for plant growth. Despite its abundance in the soil, it is scarcely bioavailable to plants due to its low solubility [81, 82]. Many soil microbes have the ability to solubilize mineral phosphorous through production of organic acids or phosphatases [12]. This functional property was displayed by 21 % of strains in this study (Fig. 4) primarily *Bacillus*, lactic acid bacteria and *Enterobacteriaceae*, consistent with previous reports [27, 82, 83]. These strains may have potential for future crop improvement.

#### Siderophore production

Like phosphorous, iron primarily exists in insoluble forms, which decreases its bioavailability to plants. Plants and microbes have the ability to overcome this problem by either lowering soil pH through organic acid production or by synthesis of siderophores, which chelate iron [12]. With respect to our findings, only 12 % of strains showed evidence of siderophore secretion, of which half belonged to the *Enterobacteriaceae*, along with three *Pseudomonas* strains (Fig. 4). These results agree with previous studies reporting the capability of soil-associated fluorescent pseudomonads to secrete iron-chelating compounds such as pyoverdine [84], as well as the secretion of a catecholate-type siderophore called enterobactin which is specific to the *Enterobateriacae* family [85].

### Extracellular enzymes

Some extracellular proteases help microbes to colonize plant roots [8], while some proteases secreted by certain strains of *Bacillus* are toxic to nematodes through cuticle degradation activity [86]. Cellulase and pectinase enzymes have similarly been shown to help degrade plant cell walls to permit entry of root-colonizing endophytes [3]. Extracellular cellulases and proteases are also used by microbes to help construct polysaccharide- and peptide-rich biofilms that help to establish the microbial community and permit attachment to host cells (e.g., rhizosphere surface, inside plants) [15]. In our study, *Bacillus and Paenibacillus* accounted for all or most stains that produced extracellular enzymes (protease, cellulase, pectinase) (Fig. 4). In particular, the most common trait of the seed associated microbiota was protease activity (46 %, 78/169 isolates), almost all of which were produced by *Bacillus* (59/83 of isolates) and *Paenibacillus* (15/37 of isolates). Proteins are a fundamental source of nitrogen in the soil, assimilated by plants through two major mechanisms: first, through root-secreted proteases, and second, by acquisition of intact proteins via endocytosis followed by protease-mediated degradation [87]. In a previous study by Johnston-Monje and Raizada [27], migration of maize seed associated bacterial endophytes to the roots and rhizosphere was observed. It is interesting to hypothesize whether the cucurbits have selected *Bacillus* microbes as part of their core seed microbiota to assist with protease-mediated nitrogen uptake and assimilation, following their migration to roots.

### A link between the cucurbit seed microbiome and the human gut microbiome?

The human gut microbiome is affected significantly by diet [88, 89]. Aside from consumption of the gourds (fruits), the seeds of some cucurbits are staple foods in diverse societies, eaten both cooked (e.g., egusi in West Africa and pepitas in Latin America) [48–50] and raw (e.g., cucumbers) [90]. As seeds are well known to be colonized by microbes [3, 27], an unexplored idea is whether either their raw consumption, or even cooked consumption (in the case of spore forming *Bacilli*), may affect the human gut microbiome [88]. The human gut microbiome is dominated primarily by two phyla (*Firmicutes* and *Bacteroidetes*), along with less represented phyla *Actinobacteria*, *Proteobacteria* and *Verrucomicrobia* (constituting 2 % of gut microbiota) [88, 89]; the *Firmicutes*, *Actinobacteria* and *Proteobacteria* similarly dominate the cucurbit seed microbiota. Based on our results, it is interesting to speculate whether the human gut microbiome is directly or indirectly, influenced by the microbiome of edible cucurbit seeds, in particular lactic acid bacteria and *Bacilli* that are well known human probiotics (Additional file 1: Table S1) [91, 92].

### The need for future metagenomic studies

A limitation of this study was that culture-independent approaches to characterize the cucurbit seed microbiome were not used. Such approaches would reveal much insight into the diversity of the seed endophytic communities that failed to grow on the used nutrient agar media. In addition, metagenomics, metatranscriptomics and metaproteomics can help in understanding the functionality of the gene pool of the seed microbiome and the putative interactions between the host and its microbiome [1]. For example, in a culture-independent study of the rice root-associated endophytic microbiome, beneficial functional traits comprising plant growth promotion and antagonism to pathogens could be predicted by metagenomic analysis of protein domains involved in metabolic processes [93].

## Conclusion

This study has revealed a cultured core microbiota associated with the seeds of seven species of the most economically important cucurbits, consisting primarily of spore-forming *Bacilli. Bacillus* was observed to be the most abundantly isolated bacterial genus with potential to contribute diverse nutrient acquisition and growth promotion activities to their hosts. The seeds may be vectors for these microbes, to help their host plants acquire nutrients and stimulate growth – results that must now be confirmed *in planta*. Such research may lead to the development of novel biofertilizers, coated onto seeds as stable spores.

## Abbreviations

ACC, 1-aminocyclopropane-1-carboxylic acid; BNF, biological nitrogen fixation; IAA, indole-3-acetic acid; LAB, lactic acid bacteria; ML, maximum likelihood; PDA, potato dextrose agar; RDP, ribosomal database project

## Additional files

Additional file 1: Table S1. Details of seed-associated cucurbit bacterial strains isolated in this study. (XLSX 166 kb) Additional file 2: Figure S1. Maximum likelihood phylogenetic tree of the total collection of putative seed-associated endophytes cultured from cucurbits, based on bacterial 16S rRNA gene sequences. Bootstrap values are indicated above the branches. (PDF 52 kb) Additional file 3: Table S2. Detailed information of in vitro phenotypic traits of cultivated cucurbit seed bacterial endophytes. (XLSX 25 kb)
