# Supplementary material for: Taxonomic and functional diversity of cultured seed associated microbes of the cucurbit family
Source: BMC Microbiol. 2016 Jun 27;16:131. doi: 10.1186/s12866-016-0743-2 (PMC4924336; doi:10.1186/s12866-016-0743-2)
Supplement: Additional file 2: Figure S1. — Maximum likelihood phylogenetic tree of the total collection of putative seed-associated endophytes cultured from cucurbits, based on bacterial 16S rRNA gene sequences. Bootstrap values are indicated above the branches. (PDF 52 kb) [file 12866_2016_743_MOESM2_ESM.pdf]

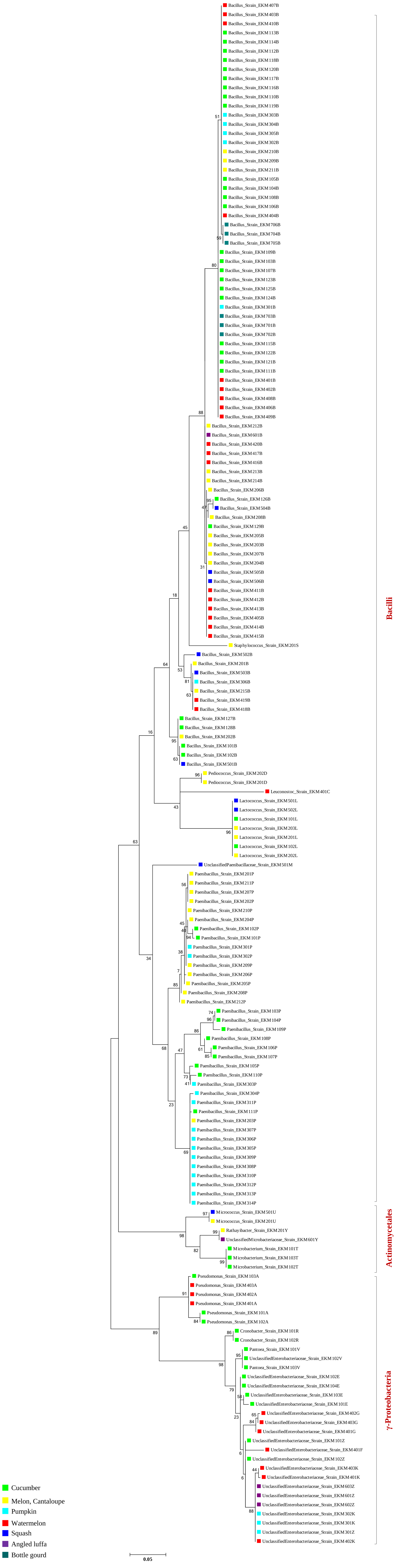
Bacillus_Strain_EKM407B
Bacillus_Strain_EKM403B
Bacillus_Strain_EKM410B
Bacillus_Strain_EKM113B
Bacillus_Strain_EKM114B
Bacillus_Strain_EKM112B
Bacillus_Strain_EKM118B
Bacillus_Strain_EKM120B
Bacillus_Strain_EKM117B
Bacillus_Strain_EKM116B
Bacillus_Strain_EKM110B
Bacillus_Strain_EKM119B
Bacillus_Strain_EKM303B
Bacillus_Strain_EKM304B
Bacillus_Strain_EKM305B
Bacillus_Strain_EKM302B
Bacillus_Strain_EKM210B
Bacillus_Strain_EKM209B
Bacillus_Strain_EKM211B
Bacillus_Strain_EKM105B
Bacillus_Strain_EKM104B
Bacillus_Strain_EKM108B
Bacillus_Strain_EKM106B
Bacillus_Strain_EKM404B
Bacillus_Strain_EKM706B
Bacillus_Strain_EKM704B
Bacillus_Strain_EKM705B
Bacillus_Strain_EKM109B
Bacillus_Strain_EKM103B
Bacillus_Strain_EKM107B
Bacillus_Strain_EKM123B
Bacillus_Strain_EKM125B
Bacillus_Strain_EKM124B
Bacillus_Strain_EKM301B
Bacillus_Strain_EKM703B
Bacillus_Strain_EKM701B
Bacillus_Strain_EKM702B
Bacillus_Strain_EKM115B
Bacillus_Strain_EKM122B
Bacillus_Strain_EKM121B
Bacillus_Strain_EKM111B
Bacillus_Strain_EKM401B
Bacillus_Strain_EKM402B
Bacillus_Strain_EKM408B
Bacillus_Strain_EKM406B
Bacillus_Strain_EKM409B
Bacillus_Strain_EKM212B
Bacillus_Strain_EKM601B
Bacillus_Strain_EKM420B
Bacillus_Strain_EKM417B
Bacillus_Strain_EKM416B
Bacillus_Strain_EKM213B
Bacillus_Strain_EKM214B
Bacillus_Strain_EKM206B
Bacillus_Strain_EKM126B
Bacillus_Strain_EKM504B
Bacillus_Strain_EKM208B
Bacillus_Strain_EKM129B
Bacillus_Strain_EKM205B
Bacillus_Strain_EKM203B
Bacillus_Strain_EKM207B
Bacillus_Strain_EKM204B
Bacillus_Strain_EKM505B
Bacillus_Strain_EKM506B
Bacillus_Strain_EKM411B
Bacillus_Strain_EKM412B
Bacillus_Strain_EKM413B
Bacillus_Strain_EKM405B
Bacillus_Strain_EKM414B
Bacillus_Strain_EKM415B
Staphylococcus_Strain_EKM201S
Bacillus_Strain_EKM502B
Bacillus_Strain_EKM201B
Bacillus_Strain_EKM503B
Bacillus_Strain_EKM306B
Bacillus_Strain_EKM215B
Bacillus_Strain_EKM419B
Bacillus_Strain_EKM418B
Bacillus_Strain_EKM127B
Bacillus_Strain_EKM128B
Bacillus_Strain_EKM202B
Bacillus_Strain_EKM101B
Bacillus_Strain_EKM102B
Bacillus_Strain_EKM501B
Pediococcus_Strain_EKM202D
Pediococcus_Strain_EKM201D
Leuconostoc_Strain_EKM401C
Lactococcus_Strain_EKM501L
Lactococcus_Strain_EKM502L
Lactococcus_Strain_EKM101L
Lactococcus_Strain_EKM203L
Lactococcus_Strain_EKM201L
Lactococcus_Strain_EKM102L
Lactococcus_Strain_EKM202L
UnclassifiedPaenibacillaceae_Strain_EKM501M
Paenibacillus_Strain_EKM201P
Paenibacillus_Strain_EKM211P
Paenibacillus_Strain_EKM207P
Paenibacillus_Strain_EKM202P
Paenibacillus_Strain_EKM210P
Paenibacillus_Strain_EKM204P
Paenibacillus_Strain_EKM102P
Paenibacillus_Strain_EKM101P
Paenibacillus_Strain_EKM301P
Paenibacillus_Strain_EKM302P
Paenibacillus_Strain_EKM209P
Paenibacillus_Strain_EKM206P
Paenibacillus_Strain_EKM205P
Paenibacillus_Strain_EKM208P
Paenibacillus_Strain_EKM212P
Paenibacillus_Strain_EKM103P
Paenibacillus_Strain_EKM104P
Paenibacillus_Strain_EKM109P
Paenibacillus_Strain_EKM108P
Paenibacillus_Strain_EKM106P
Paenibacillus_Strain_EKM107P
Paenibacillus_Strain_EKM105P
Paenibacillus_Strain_EKM110P
Paenibacillus_Strain_EKM303P
Paenibacillus_Strain_EKM304P
Paenibacillus_Strain_EKM311P
Paenibacillus_Strain_EKM111P
Paenibacillus_Strain_EKM203P
Paenibacillus_Strain_EKM307P
Paenibacillus_Strain_EKM306P
Paenibacillus_Strain_EKM305P
Paenibacillus_Strain_EKM309P
Paenibacillus_Strain_EKM308P
Paenibacillus_Strain_EKM310P
Paenibacillus_Strain_EKM312P
Paenibacillus_Strain_EKM313P
Paenibacillus_Strain_EKM314P
Micrococcus_Strain_EKM501U
Micrococcus_Strain_EKM201U
Rathayibacter_Strain_EKM201Y
UnclassifiedMicrobacteriaceae_Strain_EKM601Y
Microbacterium_Strain_EKM101T
Microbacterium_Strain_EKM103T
Microbacterium_Strain_EKM102T
Pseudomonas_Strain_EKM103A
Pseudomonas_Strain_EKM403A
Pseudomonas_Strain_EKM402A
Pseudomonas_Strain_EKM401A
Pseudomonas_Strain_EKM101A
Pseudomonas_Strain_EKM102A
Cronobacter_Strain_EKM101R
Cronobacter_Strain_EKM102R
Pantoea_Strain_EKM101V
UnclassifiedEnterobacteriaceae_Strain_EKM102V
Pantoea_Strain_EKM103V
UnclassifiedEnterobacteriaceae_Strain_EKM102E
UnclassifiedEnterobacteriaceae_Strain_EKM104E
UnclassifiedEnterobacteriaceae_Strain_EKM103E
UnclassifiedEnterobacteriaceae_Strain_EKM101E
UnclassifiedEnterobacteriaceae_Strain_EKM402G
UnclassifiedEnterobacteriaceae_Strain_EKM403G
UnclassifiedEnterobacteriaceae_Strain_EKM401G
UnclassifiedEnterobacteriaceae_Strain_EKM101Z
UnclassifiedEnterobacteriaceae_Strain_EKM401F
UnclassifiedEnterobacteriaceae_Strain_EKM102Z
UnclassifiedEnterobacteriaceae_Strain_EKM403K
UnclassifiedEnterobacteriaceae_Strain_EKM401K
UnclassifiedEnterobacteriaceae_Strain_EKM603Z
UnclassifiedEnterobacteriaceae_Strain_EKM601Z
UnclassifiedEnterobacteriaceae_Strain_EKM602Z
UnclassifiedEnterobacteriaceae_Strain_EKM302K
UnclassifiedEnterobacteriaceae_Strain_EKM301K
UnclassifiedEnterobacteriaceae_Strain_EKM301Z
UnclassifiedEnterobacteriaceae_Strain_EKM402K
Bacilli
Actinomycetales
γ-Proteobacteria
Cucumber
Melon, Cantaloupe
Pumpkin
Watermelon
Squash
Angled luffa
Bottle gourd
0.05
